# High density SNP mapping and QTL analysis for time of leaf budburst in *Corylus avellana* L.

**DOI:** 10.1371/journal.pone.0195408

**Published:** 2018-04-02

**Authors:** Daniela Torello Marinoni, Nadia Valentini, Ezio Portis, Alberto Acquadro, Chiara Beltramo, Shawn A. Mehlenbacher, Todd C. Mockler, Erik R. Rowley, Roberto Botta

**Affiliations:** 1 Dipartimento di Scienze Agrarie, Forestali e Alimentari, Università degli Studi di Torino, Grugliasco, Torino, Italy; 2 Department of Horticulture, Oregon State University, Corvallis, Oregon, United States of America; 3 Donald Danforth Plant Science Center, St. Louis, Missouri, United States of America; CIRAD, FRANCE

## Abstract

The growing area of European hazelnut (*Corylus avellana* L.) is increasing, as well as the number of producing countries, and there is a pressing need for new improved cultivars. Hazelnut conventional breeding process is slow, due to the length of juvenile phase and the high heterozygosity level. The development of genetic linkage maps and the identification of molecular markers tightly linked to QTL (quantitative trait loci) of agronomic interest are essential tools for speeding up the selection of seedlings carrying desired traits through marker-assisted selection. The objectives of this study were to enrich a previous linkage map and confirm QTL related to time of leaf budburst, using an F_1_ population obtained by crossing Tonda Gentile delle Langhe with Merveille de Bollwiller. Genotyping-by-Sequencing was used to identify a total of 9,999 single nucleotide polymorphism markers. Well saturated linkage maps were constructed for each parent using the double pseudo-testcross mapping strategy. A reciprocal translocation was detected in Tonda Gentile delle Langhe between two non-homologous chromosomes. Applying a bioinformatic approach, we were able to disentangle ‘pseudo-linkage’ between markers, removing markers around the translocation breakpoints and obtain a linear order of the markers for the two chromosomes arms, for each linkage group involved in the translocation. Twenty-nine QTL for time of leaf budburst were identified, including a stably expressed region on LG_02 of the Tonda Gentile delle Langhe map. The stability of these QTL and their coding sequence content indicates promise for the identification of specific chromosomal regions carrying key genes involved in leaf budburst.

## Introduction

*Corylus avellana* L. is the major species of interest for alimentary use within the Betulaceae family. The worldwide production of in-shell hazelnuts is estimated to be 835,000t (average 2012–2016). The growing area has increased from 607,500 ha in 2010 to 661,500 ha in 2016 (+8.9%), [1] due to a worldwide growth in demand, as the health benefits of nut consumption is increasingly recognized [2] and the marketing of processed products has enlarged, with most of the production used by the confectionary industry. The world hazelnut consumption is estimated to be about 390,000 t of shelled nuts (average 2011–2015) [3].

The natural distribution of *C*. *avellana* is restricted to the northern hemisphere. Traditional hazelnut-producing regions have mild, humid winters and cool summers (Mediterranean-type climates), and are located at middle latitudes (40–45°N). Although Turkey and Italy remain the major producing countries (80% of the world crop), hazelnut cultivation has spread in recent years to new growing areas including the southern hemisphere.

Hazelnut cultivation is based on a small number of traditional cultivars selected for their adaptability to the soil and climate of their areas of origin, and the needs of the processing industry. The expansion of hazelnut cultivation, ongoing climate changes, and the vulnerability of a crop due to the limited number of good cultivars [4,5] require the breeding of improved cultivars, aided by advanced breeding strategies. Knowledge of the genetic structure of the species is vital for managing these threats, and facilitates the selection of productive genotypes improved for nut quality, adapted to specific environments, and tolerant or resistant to pest and diseases.

In hazelnut, the conventional breeding process is slow, due to the length of juvenile phase and the high heterozygosity level. The development of genetic linkage maps and the identification of molecular markers tightly linked to QTL (quantitative trait loci) of agronomic interest would speed up the selection of seedlings carrying desired traits through marker-assisted selection (MAS). Moreover, the identification of QTL and genomic regions associated with trait variation would allow identification of key genes and linked markers, leading to the identification of candidate genes.

In recent years, single nucleotide polymorphisms (SNPs) have been extensively used for high-density genetic mapping, since they allow identification of QTL with greater precision than it is possible with other types of molecular markers [6,7]. SNPs are the most abundant type of marker in plant genomes [8], typically occurring at frequencies of one per 100–500 bp [9]. They have a low mutation rate and are evolutionary stable from generation to generation across the genome [10]. SNP markers have shown their full potential with the advent of next generation sequencing (NGS) technologies. Elshire et al. (2011) [11] developed a technique called genotyping-by-sequencing (GBS), which reduced genome complexity through the use of restriction enzymes. GBS can be used for various applications including discovery of SNPs in large quantities. High density genetic maps and QTL mapping using SNPs discovered by the GBS approach have been developed in several species such as barley and wheat [12], maize [13], peach [14], chickpea [15], pepper [16], sunflower [17] and rubber tree [9].

Hazelnut is a diploid species with 11 pairs of chromosomes (2n = 2x = 22) and an estimated genome size of 378 Mb (http://www.cavellanagenomeportal.com). Figures observed during meiosis indicate the presence of reciprocal translocations in a few cultivars, including Tonda Gentile delle Langhe, Barcelona and Tonda di Giffoni [18,19]. Such translocations would alter the segregation of markers in progenies used for mapping purposes, leading to ‘pseudo-linkage’ and the creation of a single linkage group that included a mix of markers from the chromosomes involved in the phenomenon [20,21].

In hazelnut, advanced genomic tools to aid in breeding efforts are under development. Nowadays these tools include a transcriptome [22] and a genome assembly of the eastern filbert blight resistant cultivar Jefferson (http://www.cavellanagenomeportal.com). Other genomic resources include the resequenced whole genomes of seven additional hazelnut accessions, which have been used to investigate genetic diversity and develop new simple sequence repeat (SSR) markers (http://www.cavellanagenomeportal.com). Also available is a genetic map for the cross OSU 252.146 × OSU 414.062 constructed with random amplified polymorphic DNA (RAPD) and SSR markers [23] to which additional SSR markers were added by Gürcan and Mehlenbacher (2010) [24], Gürcan et al. (2010) [25], Colburn et al. (2017) [26] and Bhattarai and Mehlenbacher (2017) [27]. The addition of GBS markers [28] resulted in an improved high-density map. Beltramo et al. (2016) [29] developed a new linkage map with SSR markers and performed a QTL analysis for vigor, sucker habit, and time of budburst. Recently, Özturk et al. (2017) [30] applied association mapping for nut and kernel traits in Slovenian hazelnut germplasm.

The purpose of our study was: (i) to develop SNP markers, discovered by a GBS approach, to saturate the genetic SSR-based map constructed by Beltramo et al. (2016) [29], (ii) to obtain linkage maps using a bioinformatic approach to disentangle the two linkage groups of Tonda Gentile delle Langhe that had been merged into a single large ‘pseudo-linkage’ group, due to the occurrence of a reciprocal translocation, (iii) to confirm QTL associated with the time of leaf budburst, described in Beltramo et al. (2016) [29], and (iv) to detect candidate genes involved in regulation of the time of leaf budburst.

## Materials and methods

### Plant materials

A progeny of 275 F_1_ individuals was obtained by crossing Tonda Gentile delle Langhe (syn. Tonda Gentile, Tonda Gentile Trilobata, hereafter TGdL) as the female parent with Merveille de Bollwiller (syn. Hall’s Giant, hereafter MB) as the male one. The controlled cross was performed in February 2008 as reported by Beltramo et al. 2016 [29]; the 275 seedlings and three individuals obtained from rooted suckers of each of the two parents were planted in November 2010 and evaluated over six years (2011–2016). The field is located on the campus of the University of Torino, Department of Agricultural, Forest and Food Sciences (45°07’N; 7°58’E; 293 m a.s.l.). The seedlings and parents were planted at a spacing of 4 x 4 m and trained in an open vase system.

### DNA isolation and discovery of SNPs

The GBS approach [11] was used to discover SNP markers. The mapping population was a set of 213 (of the 275) F_1_ individuals of the progeny TGdL x MB, including the 163 plants previously analysed by Beltramo et al. (2016) and 50 new individuals [29]. Only the plants situated at the edge of the experimental field were excluded.

Total genomic DNA was extracted from young leaves collected in the spring using a CTAB method [31]. Quantity and quality of extracted DNA were determined by the Qubit assay (Thermo Fisher Scientific) and by electrophoresis on 1% agarose gel and comparison against a Lambda DNA/EcoRI + HindIII marker (Thermo Fisher Scientific). In October 2014, approximately 3 μg of genomic DNA from each individual and the two parents were sent to the Genomic Diversity Facility at Cornell University—Institute of Biotechnology (USA) (http://www.biotech.cornell.edu/brc/genomic-diversity-facility) for GBS. Briefly, GBS libraries were constructed in 96-plex by digesting genomic DNA with the restriction enzyme *Ape*KI, a five-base cutter (5’ GCWGC 3’), followed by ligation of a barcode adaptor and a common Illumina sequencing adaptor to the fragmented DNA. The resulting libraries were run through an Illumina Hiseq2500 flow cell for sequencing.

### Sequence analysis

Raw reads were analyzed with Scythe (https://github.com/vsbuffalo/scythe) to filter out contaminant substrings and Sickle (https://github.com/najoshi/sickle) to remove reads with poor quality ends (Q<30). Illumina reads were de-multiplexed on the basis of the barcode sequences (4-8bp). Alignment to the reference hazelnut genome (http://www.cavellanagenomeportal.com) was carried out using BWA aligner [32] (i.e., mem command) with default parameters and avoiding multiple-mapping reads. SNP mining was conducted by adopting a Samtools-based pipeline [33]. Homozygous/heterozygous SNP/Indel calls were considered only with a phred-scaled genotype likelihood equal to zero. Loci with mean deepness (DP) less than 10 were filtered out through the vcftools pipeline (http://vcftools.sourceforge.net). A catalogue of candidate high quality SNPs was produced by subdividing them into five SNP types according to their polymorphism between the parents TGdL and MB and within the F_1_ population. The SNPs were named starting with Sc_AJ (*Scaffold Avellana Jefferson*) followed by two numbers representing the number of the scaffold and the SNP position on the scaffold.

### SSR analyses

A set of 24 microsatellite primer pairs [24,25,34,35] were used to amplify the two parents and 213 seedlings. PCR reactions were performed as described by Beltramo et al. (2016) [29]. Amplification products were analysed on a 3130 Genetic Analyzer capillary sequencer (Applied Biosystems, USA). Allele sizes were called using GeneMapper 4.0 software (Applied Biosystems).

### Linkage mapping

Independent framework linkage maps were constructed for each parent using the double pseudo-testcross mapping strategy [36] and JoinMap v4.0 [37]. Two separate data sets were assembled with only SNP markers in testcross configuration (expected segregation ratio of 1:1): maternal testcross markers segregating only in TGdL and paternal testcross markers segregating only in MB. SSR markers with three or four alleles (one parent ab, the other either ac or cd), and an expected segregation ratio of 1:1:1:1, were scored as 1:1 markers according to the parental origin of the segregating alleles, and included in either the maternal or paternal data set. Goodness-of-fit between observed and expected segregation ratios was assessed using the χ^2^ test. Markers fitting a Mendelian pattern closely associated with a χ^2^ value ≤ χ^2^_α = 0.1_ or with only a minor deviation (χ^2^_α = 0.1_< χ^2^ ≤ χ^2^_α = 0.01_) were used for map construction, provided that their inclusion did not alter the local marker order. Loci suffering from significant segregation distortion (χ^2^ > χ^2^_α = 0.01_) were excluded. The similarity of loci option of JoinMap was used to identify perfectly identical markers (similarity value = 1.000), which would map to exactly the same position. To reduce the load of calculation effort, only one representative of each group of similar loci was used for mapping.

For both maps, LGs were established based on a threshold logarithm of odds (LOD) ratio of 12.0. To determine marker order within a linkage group (LG), the JoinMap parameters were set at Rec = 0.40, LOD = 1.0 and Jump = 5. Map distances were converted to centiMorgans (cM) using the Kosambi mapping function [38]. Linkage maps were drawn using MapChart 2.2 software [39], and markers deviating in their segregation only marginally from the expected Mendelian ratio are presented with one (χ^2^_α = 0.1_ < χ^2^ ≤ χ^2^_α = 0.05_) or two (χ^2^_α = 0.05_ < χ^2^ ≤ χ^2^_α = 0.01_) asterisks. According to the map locations of the SSR markers [29], LG of the female and male maps were respectively named TGdL_01 to TGdL_11 and MB_01 to MB_11, respectively.

A ‘pseudo-linkage’ found between markers segregating for the TGdL parental line indicated the presence of a reciprocal translocation between linkage groups. The strategy reported by Farré et al. (2011) [21] was adopted for the construction of the TGdL map: (i) identification and disentanglement of the ‘pseudo-linkage’ via principal co-ordinate (PCO) analysis, carried out to clarify the multi-dimensional relationships between the involved TGdL linkage group markers; (ii) sorting F1 individuals into translocated and normal groups; (iii) classification of markers into the classes of close to or more distant from the translocation sites and (iv) construction of linkage groups beginning with the most distant sections of LGs involved in the translocation. Marker similarity was assessed via a simple matching coefficient, equal to one minus the recombination frequency; calculations were performed using NTSYS v2.10 [40].

### Phenotypic traits and QTL analysis

The progeny segregated for several phenological traits, including time of leaf budburst (lb), which was recorded across 5 years (2012–2016) as the stage of first leaf appearance out of the bud (“stage C1”) [41]. Time of budburst was also expressed using the UPOV (1979) [42] descriptors from very early (1) to very late (9). Population means and standard deviations were calculated, and distribution histograms drawn, using IBM SPSS Statistics 24 (New York, USA).

The two separate parental maps were used to assign putative QTL locations by performing both the simple interval mapping (SIM) [43] and multiple QTL mapping (MQM) [44] procedures implemented within MapQTL 5 software [45]. Putative QTLs were first identified using interval mapping, after which one linked marker per putative QTL was treated as a co-factor to represent genetic background control in the approximate multiple QTL model. For the MQM, a backward elimination procedure was applied to select the appropriate co-factors (e.g. significantly associated with each trait at *p* < 0.02). A mapping step size of 1 cM was used for both the SIM and MQM analyses. Log of odds (LOD) thresholds for genome-wide (P < 0.05) were empirically determined for the trait using the PERMUTATION test of MapQTL with 1,000 iterations [46]. Only those QTL associated with a LOD greater than either the genome-wide thresholds were considered, and 1-LOD support intervals were determined for each LOD peak [47]. First, the non-parametric Kruskal–Wallis (KW) test was employed to detect association between markers and traits individually. In a second step, interval mapping (IM) analysis was performed to select markers significantly associated with the trait to constitute an initial set of cofactors. A backward elimination procedure was applied to the initial set of cofactors. Only significant markers at P < 0.02 were used as cofactors in the multiple QTL method (MQM) [44] analysis for QTL detection. Based on the permutation tests, a threshold LOD value of 3.1 was used to declare the presence of a QTL.

The proportion of the overall phenotypic variance (PV) associated with each QTL was estimated from the MQM model. Each QTL was designated by “lb”, followed by the relevant linkage group (LG) and a suffix indicating the year of its expression. For example, “lb_TGdL_02_13” indicates the QTL underlying time of leaf budburst on the TGdL map, linkage group 02, and identified by analysing data from the year 2013.

## Results

### SNP discovery using genotyping-by-sequencing

The GBS approach generated a total of 46.2 Gb of DNA sequences. The raw data were demultiplexed according to the barcode sequences, trimmed by eliminating the sequences of the barcodes/adapters, and low-quality bases were removed. The result was an average 2,028,916 reads per seedling sample, and 2,138,569 and 2,109,994 reads for the parents, TGdL and MB, respectively. Cleaned reads with quality scores > 30 were mapped against the hazelnut genome (36,641 scaffolds) and high quality mapped sequences (more than 10 reads/locus) covered approximately 2,060,957 Mb, representing 0.6% of the genome sequence. Relative to the referenced sequences, 9,999 single nucleotide substitutions were identified, with a frequency of one SNP every 206 nucleotides. The SNPs were of five types (Table 1), and a complete list of all SNPs identified in this study is reported in S1 File. Markers were coded as "Sc.AJ_x_y", where "Sc.AJ" means Scaffold Avellana Jefferson, "x" is the number of the scaffold to which the marker sequence aligns and "y" is the position of the SNP marker within the scaffold.

**Table 1:** SNP types identified in this study through genotyping-by-sequencing.

SNP type (parental state)	N°	Expected segregation
**I) Both parents homozygous, monomorphic**	1,954	Not segregating (all progeny homozygous)
**II) Both parents homozygous, polymorphic**	702	Not segregating (all progeny heterozygous)
**III) Both parents heterozygous, monomorphic**	2,537	1:2:1
**IV) TGdL heterozygous, MB homozygous**	2,338	1:1
**V) TGdL homozygous, MB heterozygous**	2,468	1:1
**Total**	9,999	

### Marker data for linkage analysis

Of the 7,343 segregating SNP markers discovered, 2,537 SNP markers were heterozygous in both parents, while 2,338 and 2,468 were heterozygous only in TGdL and MB, respectively (Table 1). Only SNP markers segregating in only one of the parents were retained for map construction by treating the F_1_ population as a backcross. Within the TGdL-specific markers, 408 were excluded from further map construction as they showed highly significant distortion from the expected 1:1 ratio, and 264 were excluded because they showed identical segregation patterns (100% similarity with other loci). Similarly, 607 SNP markers segregating from MB showed highly significant distortion and 231 showed identical segregation patterns and so were discarded prior to the construction of the linkage map. Therefore, 1,666 and 1,630 SNP markers were available for TGdL and MB mapping, respectively. To these SNP markers, we added 20 and 19 segregating SSR loci (for TGdL and MB, respectively). In order to produce more accurate linkage maps, a further stringent selection was applied, considering only markers grouped at LOD of 12.0 (1,576 and 1,562 markers for TGdL and MB, respectively). In addition, only one marker per scaffold was used for the framework map construction and, consequently, 340 and 351 SNP markers for TGdL and MB, respectively, were considered accessory markers. A full list of these accessory markers is provided (S2 File). Finally, a set of 1,236 and 1,211 non-redundant markers (SNPs and SSR) were used for constructing the maps of TGdL and MB, respectively.

### Preliminary linkage analysis and recombination around the TGdL translocation breakpoints

**Fig 1:**
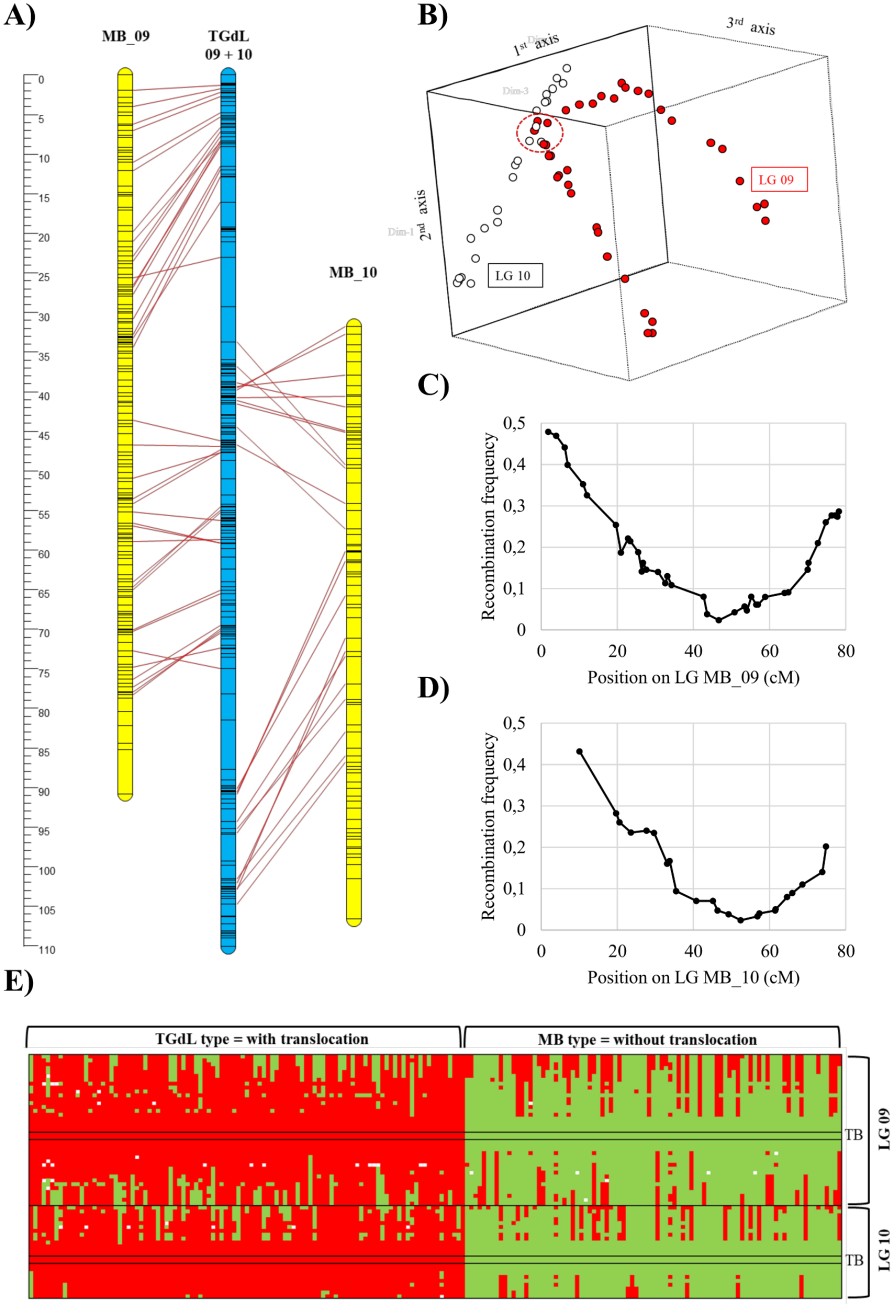
Clarification of reciprocal translocation in Tonda Gentile delle Langhe (TGdL). (A) Schematic linkage groups MB_9 and MB_10 aligned to the entangled LG for TGdL. (B) Three-axis principal coordinate analysis of the TGdL markers of LG_9 (solid red) and _10 (open circles), related to the MB linkage groups. The red circle enclose markers of LGs TGdL_9 and TGdL_10 that are adjacent to the translocation discontinuities; thus they are separated in only very few recombinations. (C) Frequency of TGdL recombination between LG_9 markers and marker Sc.AJ_04559_13309 of LG_10, relative to their position on MB_9. (D) Frequency of TGdL recombination between markers of LG_10 with marker Sc.AJ_01333_17769 of LG_09, against their position on MB_10. (E) Genotypes of individual markers on TGdL_09 and _10 according to the MB map. Individuals were classified according to their alleles at the translocation breakpoints: the TGdL type (red), which exhibits the translocation, and the MB type (green) which has the pre-translocation chromosome architecture.

For initial linkage analysis, LOD thresholds in the range 4–12 were used for grouping the markers, resulting in the separation of the MB markers into 11 clear linkage groups (the haploid number of the species). In contrast, the TGdL markers were placed in only 10 LGs. Markers located on scaffolds common to the two maps were used for detection of homologue LGs. For nine of the linkage group pairs a one-to-one correspondence was found between the TGdL and MB maps; however a single TGdL group containing 193 markers matched two groups of the MB map (MB_09 and MB_10). This “pseudo-linkage” of TGdL markers may be explained by a reciprocal translocation between TGdL_09 and TGdL_10. Fig 1A displays the TGdL linkage group with 193 markers drawn with the regression mapping algorithm and aligned with MB_09 and MB_10 ones, on the basis of markers developed on 38 and 22 common scaffolds respectively. Repeating the analysis using the maximum likelihood algorithm (data not reported) gave a different LG, demonstrating that these markers could not be aligned in a unique sequence. Principal coordinate analysis was used to visualize the relationships between the common markers and Fig 1B displays the first three principal axes showing the positions of these markers. The percentages of variation explained by the first three axes were 29.9, 17.4 and 14.7, respectively. Markers of LGs TGdL_09 and 10 are displayed in red and white respectively, according to their position on the MB linkage groups. Fig 1B shows that LGs TGdL_09 and 10 coincide at one position (shown by a red dashed circle). In the PCA map the chromosome arms split from this position, and from one another, demonstrating that the translocation breakpoints occur within that point. Recombination at the breakpoints sites was assessed by estimation of recombination frequencies between the markers for TGdL LGs 09 and 10. We used the MB map to allocate markers to linkage groups and place them in the correct sequence. Marker pair Sc.AJ_01333_17769 (LG09) and Sc.AJ_04559_13309 (LG10) had the lowest recombination frequency, 0.023, (5 recombinants out of 213 F1 individuals). Fig 1C and 1D show the marker recombination frequencies and their corresponding positions on the MB map. As expected, markers near the translocation breakpoints have negligible recombination frequencies. Following the method of Farré et al. (2011) [21], the F_1_ population was split into two subpopulations depending on the markers lying near the translocation breakpoint, one having alleles identical to TGdL, the other to MB. In the first subpopulation, the chromosomal arrangement is that of TGdL (9a-10b, 10a-9b), while the second subpopulation has the MB arrangement (9a-9b, 10a-10b). The assignment of the 213 F_1_ individuals into the two subpopulations was based on the data of markers Sc.AJ_01333_17769 and Sc.AJ_04559_13309 (Fig 1E). First, the markers were divided into linkage groups 09 and 10, subsequently sorted vertically according to their position within each of the linkage groups based on the MB map. Following this, individuals were sorted according to the origin of their allele on marker Sc.AJ_01333_17769; results were similar when the same process was carried out based on Sc.AJ_04559_13309. Assignment into these two subpopulations was not exact; 20 individuals were eliminated due to conflicting chromosomal combinations in the region up to 5 cM from the deduced translocation breakpoints.

The two subpopulations were considered separately in order to group TGdL markers of the chromosomes involved in the reciprocal translocation. Subpopulation 1 has the TGdL allele near to or at the translocation breakpoint and contained 101 seedlings while subpopulation 2, with the MB allele near the translocation breakpoint, contained 92 seedlings. The two subpopulations were subjected to independent linkage analyses allowing the sub-division of the previous “pseudo-linked” markers into four well separated groups (at LOD>4): two of them, designated TGdL_09a and 09b, included markers in common with MB_09, and the other two (TGdL_10a and 10b) with markers in common with MB_10. In an F1 population, testcross markers are expected to segregate in a 1:1 ratio. However, in both these subpopulations, markers close to the translocation breakpoints showed severe segregation distortion. In general these markers were discarded as we were unable to allocate them to any specific TGdL linkage group. Exceptions were markers found on both MB_09 and 10, which could be allocated taking into account lower nearest-neighbor stress. Finally, the TGdL_09a, 09b, 10a and 10b were developed by analysing separately the four groups of markers and the whole F_1_ seedling population.

### Map construction

**Fig 2:**
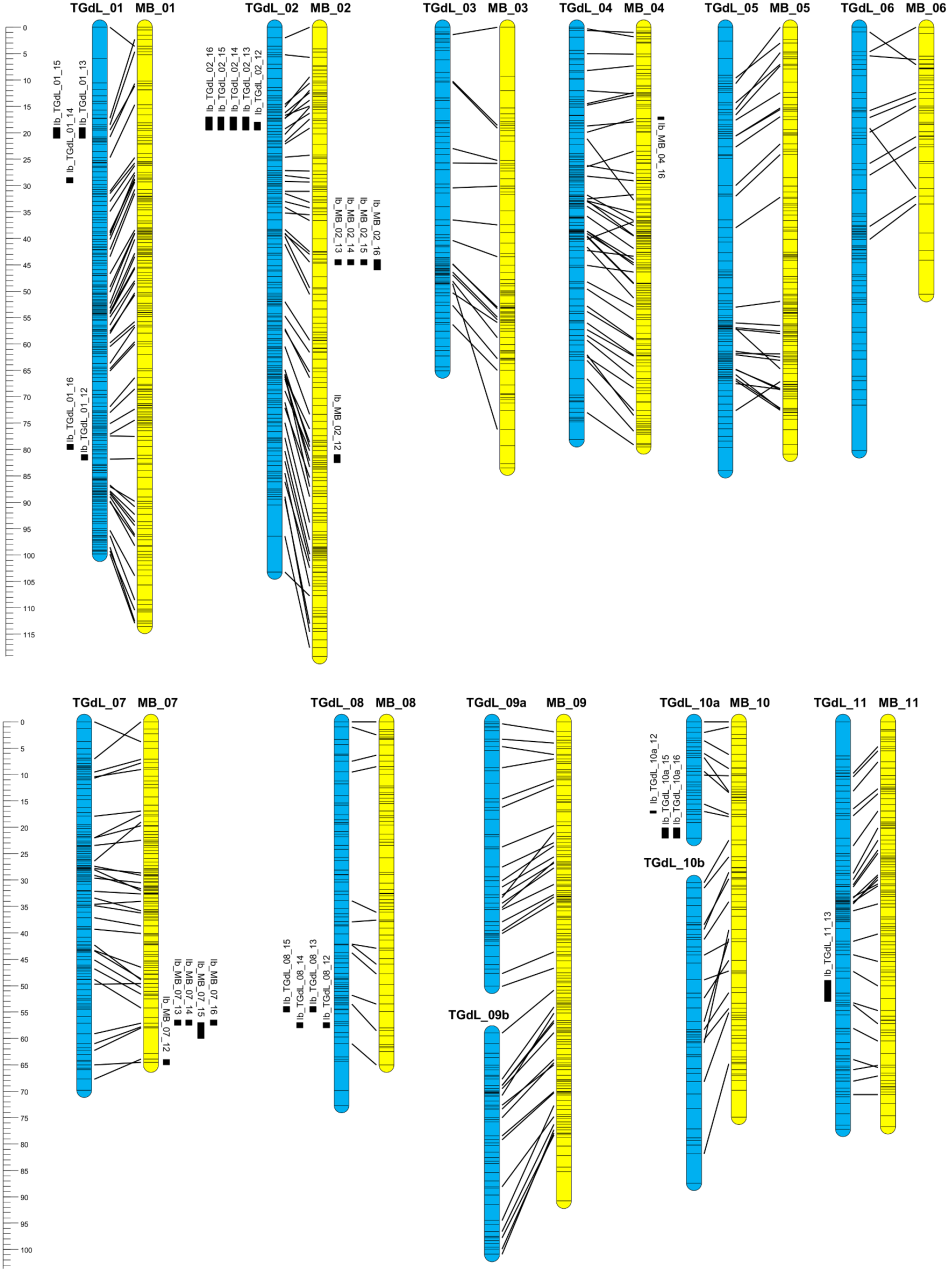
Genetic maps of the *Corylus avellana* cultivars Tonda Gentile delle Langhe (TGdL) and Merveille de Bollwiller (MB). TGdL (female parent, blue LGs on the left) and MB (male parent, yellow LGs on the right), aligned on the base of markers developed on common scaffolds, and location of QTL responsible for time of leaf budburst. Loci mapped in repulsion phase are flanked by an “-r”. The left rulers expressed the length of the LGs and the QTL position in cM.

Two well saturated genetic maps were developed (Fig 2 and S3 File). From the 1,236 markers (1,216 SNP markers and 20 SSR markers) segregating from TGdL, a map was generated which consisted of 13 LGs, for a total length of 900.4 cM, with a mean inter-marker distance of 0.8 cM (Table 2). The LG length ranged from 22.1 cM (TGdL_10a) to 103.2 cM (TGdL_02). The number of markers per chromosome was the highest in TGdL_01 (205) and the lowest in TGdL_10a (29), with an average of 95.1 markers per linkage group. The majority (77.3%) of map intervals were less than 1 cM; only eight gaps > 5cM were present. A total of 111 markers showed marginally distorted segregation and mapped to all LGs (32 at α = 0.05; 79 at α = 0.01).

**Table 2:** Characteristics of Tonda Gentile delle Langhe (TGdL) and Merveille de Bollwiller (MB) linkage maps.

Tonda Gentile delle Langhe					Merveille de Bollwiller				
Linkage group	Size (cM)	N° of markers	Marker density	Gaps (>5 cM)	Linkage group	Size (cM)	N° of markers	Marker density	Gaps (>5 cM)
**TGdL_01**	99.1	205	0.5	1	**MB_01**	113.5	172	0.7	0
**TGdL_02**	103.2	166	0.6	2	**MB_02**	119.2	156	0.8	0
**TGdL_03**	65.1	83	0.8	0	**MB_03**	83.5	79	1.1	1
**TGdL_04**	78.1	128	0.6	0	**MB_04**	79.5	144	0.6	0
**TGdL_05**	84.0	108	0.8	1	**MB_05**	80.9	115	0.7	0
**TGdL_06**	80.2	69	1.2	1	**MB_06**	50.6	39	1.3	2
**TGdL_07**	69.8	124	0.6	0	**MB_07**	64.5	107	0.6	0
**TGdL_08**	72.7	76	1.0	1	**MB_08**	65.0	67	1.0	0
**TGdL_09a**	50.1	47	1.1	0	**MB_09**	90.8	148	0.6	1
**TGdL_09b**	41.9	54	0.8	0					
**TGdL_10a**	22.1	29	0.8	0	**MB_10**	74.9	78	1.0	1
**TGdL_10b**	56.9	45	1.3	1					
**TGdL_11**	77.2	102	0.8	1	**MB_11**	76.7	106	0.7	0
**Average**	69.3	95.1	0.8	0.6	**Average**	81.7	110.1	0.8	0.5
**Total**	900.4	1,236		8	**Total**	899.1	1,211		5

Size in cM, number of markers, marker density and gaps > 5cM for TGdL and MB linkage maps.

The 1,211 markers (1,192 SNP markers and 19 SSR markers) segregating in the male parent (MB) were used to generate maps of 11 LGs, covering a total length of 899.1 cM, with a mean inter-marker distance of 0.8 cM (Table 2). The LG length ranged from 50.6 cM (MB_06) to 119.2 cM (MB_02). The number of markers per chromosome was highest in MB_01 (172) and lowest in MB_06 (39), with an average of 110.1 markers per linkage group. The majority (77.1%) of map intervals were less than 1 cM; only five gaps > 5cM were present. A total of 70 markers showed marginally distorted segregation and mapped to all LGs (16 at α = 0.05 and 54 at α = 0.01).

The presence of markers on common scaffolds between the two maps allowed identification of the corresponding LGs as well as their one-to-one correspondence, and the identification of homologous regions (Fig 2 and S3 File). The number of common scaffolds per homologous LG ranged between 11 and 52 for a total number of 330.

### Phenotyping and QTL identification for time of leaf budburst

**Fig 3:**
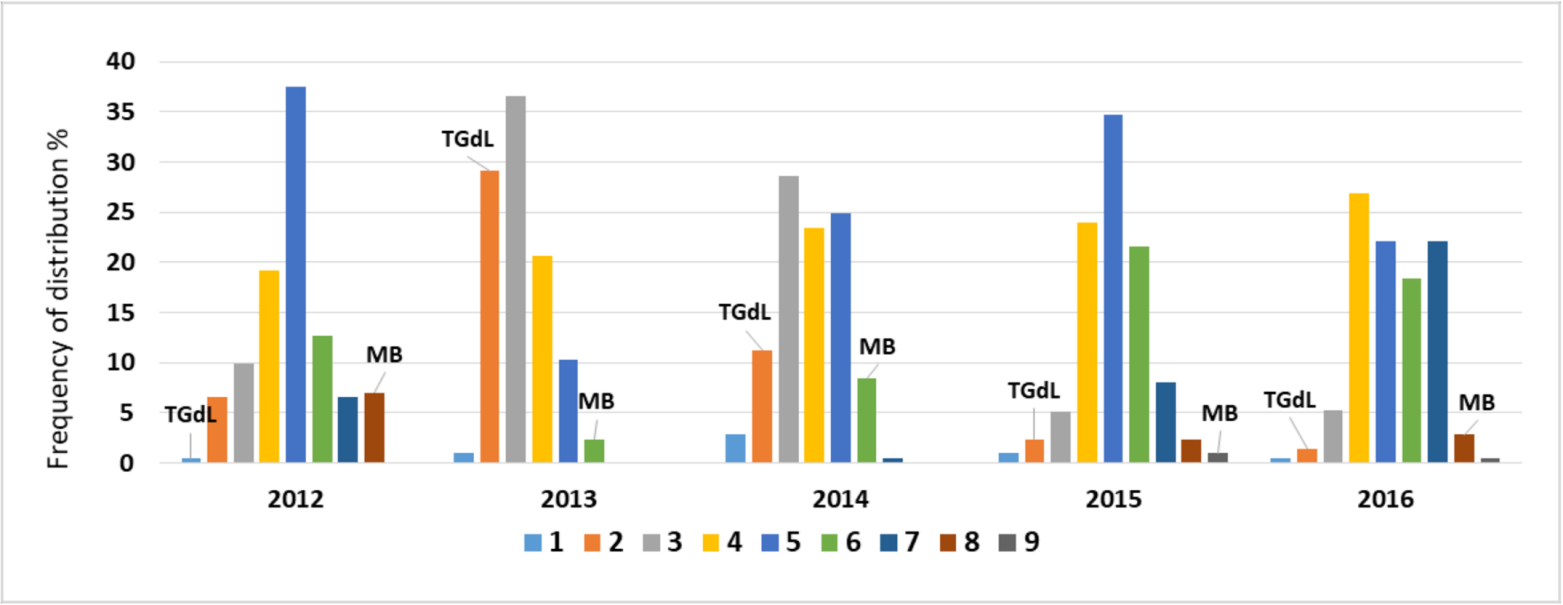
Frequency distributions (%) of time of leaf budburst for the Tonda Gentile delle Langhe (TGdL) x Merveille de Bollwiller (MB) progeny in years 2012 to 2016. Data are grouped in classes from 1 = very early to 9 = very late. Means for the parents TGdL and MB are shown for each histogram.

The phenotypic data and statistical values for time of leaf budburst are summarized (Table 3). There were significant (*p* < 0.1) phenotypic differences between TGdL and MB for time of budburst, with TGdL having an earlier budburst (ranging from class 1 to 2) than MB (ranging from class 6 to 9). The distribution of F_1_ progeny phenotypes was intermediate between the two parents and normally distributed, highlighting the polygenic control of the trait. A moderate transgression level was observed (Fig 3).

**Table 3:** Phenotypic data and statistical values for time of leaf budburst.

Year	Parent means ± SD		F_1_ population	Range	SE	Skewness	SE	Kurtosis	SE
	TGdL	MB	mean ± SD						
**2012**	1.0 ± 0.0	8.0 ± 0.0	4.86 ± 1.51	1.0–8.0	0.10	0.125	0.167	-0.000	0.332
**2013**	2.0 ± 0.0	6.0 ± 0.0	3.17 ± 1.07	1.0–6.0	0.07	0.593	0.167	-0.229	0.332
**2014**	2.0 ± 0.0	6.0 ± 0.0	3.84 ± 1.27	1.0–7.0	0.09	-0.054	0.167	-0.612	0.332
**2015**	2.0 ± 0.0	9.0 ± 0.0	5.03 ± 1.31	1.0–9.0	0.09	0.015	0.167	0.894	0.332
**2016**	2.0 ± 0.0	8.0 ± 0.0	5.30 ± 1.41	1.0–9.0	0.10	-0.052	0.167	-0.515	0.332

Parent means (±SD), F1 population mean (±SD), population range (±SE), values of Skewness and Kurtosis (±SE) for time of leaf budburst trait (rated from 1 = very early to 9 = very late)

QTL analysis was performed in each season (2012–2016) and for each parent (TGdL and MB). The QTLs that explained more the 10% of the phenotypic variance (PV) are hereafter referred to as ‘major’ QTL. Overall, 29 QTL for time of leaf budburst were identified. On the female map, 18 marker-trait associations were identified and mapped onto 6 QTL cluster regions dispersed on 5 of the 13 LGs: TGdL_01 (2 QTL regions), 02, 08, 10a and 11 (Table 4). Of the QTLs detected on the female map (Table 4 and Fig 2), two were expressed every year (2012–2016). The first of these mapped to TGdL_01, in two different regions, and explained from 3.0 to 6.3% of PV, and the second mapped to TGdL_02 where it explained from 31.4 to 54.6% of PV (a ‘major’ QTL), associated with an additive effect from 1.4 (2013) to 2.1 (2016) days, and with an associated LOD score that ranged from 23.7 (2012) to 45.3 (2016) (S4 File). One QTL was detected on TGdL_08 in all years except 2016 and explained 4.0 to 4.4 of PV. One QTL was detected on TGdL_10a in three years (2012, 2015 and 2016), explaining 4.4 to 6.4% of PV, while one QTL was only detected on TGdL_11 in year 2013 and explained 3.9% of PV.

**Table 4:** QTL detected in the female (TGdL) and male (MB) mapping population for time of leaf budburst (*lb*).

**LG**	**QTL**	**2012**						**2013**					
		**GW**	**cM**	**LOCUS**	**LOD**	**PV**	**Additive**	**GW**	**cM**	**LOCUS**	**LOD**	**PV**	**Additive**
01	**lb_TGdL_01 (A)**	3.3	-	-	-	-	-	3.1	20.8	Corav1231	6.5	6.3	-0.54
01	**lb_TGdL_01 (B)**		81.8	00364_36890	5.3	5.7	0.72		-	-	-	-	-
02	**lb_TGdL_02**		19.2	AJ417975b	23.7	31.4	-1.72		19.2	AJ417975b	32.6	43.2	-1.43
08	**lb_TGdL_08**		57.1	03460_19364	4.1	4.4	-0.65		54.6	00542_41715	4.5	4.3	-0.45
10a	**lb_TGdL_10a**		17.1	00361_58830	4.1	4.4	-0.65		-	-	-	-	-
11	**lb_TGdL_11**		-	-	-	-	-		51.4	07109_9866	4.1	3.9	0.43
02	**lb_MB_02 (A)**	3.1	-	-	-	-	-	3.1	44.5	01881_28048	4.0	7.7	0.59
02	**lb_MB_02 (B)**		82.1	08587_2729	3.6	7.0	0.80		-	-	-	-	-
04	**lb_MB_04**		-	-	-	-	-		-	-	-	-	-
07	**lb_MB_07**		64.5	00002_249778	3.3	6.3	0.76		57.1	04949_1954	3.2	6.2	0.53
**LG**	**QTL**	**2014**						**2015**					
		**GW**	**cM**	**LOCUS**	**LOD**	**PV**	**Additive**	**GW**	**cM**	**LOCUS**	**LOD**	**PV**	**Additive**
01	**lb_TGdL_01 (A)**	3.1	29.1	02104_20297	3.6	3.2	-0.46	3.0	20.8	Corav1231	5.8	6.0	-0.65
01	**lb_TGdL_01 (B)**		-	-	-	-	-		-	-	-	-	-
02	**lb_TGdL_02**		19.2	AJ417975b	35.9	46.3	-1.74		19.2	AJ417975b	25.5	33.2	-1.53
08	**lb_TGdL_08**		57.1	03460_19364	4.7	4.2	-0.53		54.4	10607_3880	3.9	4.0	-0.53
10a	**lb_TGdL_10a**		-	-	-	-	-		22.1	00502_20042	4.6	4.7	-0.58
11	**lb_TGdL_11**		-	-	-	-	-		-	-	-	-	-
02	**lb_MB_02 (A)**	3.2	44.5	01881_28048	3.3	4.1	0.51	3.0	44.5	01881_28048	3.3	6.4	0.67
02	**lb_MB_02 (B)**		-	-	-	-	-		-	-	-	-	-
04	**lb_MB_04**		-	-	-	-	-		-	-	-	-	-
07	**lb_MB_07**		57.1	04949_1954	3.8	3.6	0.48		58.0	21507_1735	3.1	3.1	0.65
**LG**	**QTL**	**2016**											
		**GW**	**cM**	**LOCUS**	**LOD**	**PV**	**Additive**						
01	**lb_TGdL_01 (A)**	3.2	-	-	-	-	-						
01	**lb_TGdL_01 (B)**		79.5	KG857	5.7	4.3	0.59						
02	**lb_TGdL_02**		19.2	AJ417975b	45.3	54.6	-2.10						
08	**lb_TGdL_08**		-	-	-	-	-						
10a	**lb_TGdL_10a**		22.1	00502_20042	8.2	6.4	-0.72						
11	**lb_TGdL_11**		-	-	-	-	-						
02	**lb_MB_02 (A)**	3.1	44.7	17921_4014	3.1	4.0	0.57						
02	**lb_MB_02 (B)**		-	-	-	-	-						
04	**lb_MB_04**		17.3	04654_12942	3.6	4.9	0.64						
07	**lb_MB_07**		57.1	04949_1954	3.5	6.8	0.74						

Each QTL name is given by the abbreviated acronym of the trait followed by the LG number. The table indicates genome-wide LOD Thresholds (GW) as determined by a permutation test at p ≤ 0.05, the closest linked marker (Locus) and their map position in cM, the estimated LODs at the QTL peak (LOD), the proportions (%) of the total phenotypic variance (PV) explained and the parental allelic contribution (Additive).

In the male map, 11 marker-trait associations were identified and mapped onto 4 QTL cluster regions dispersed on 3 of the 11 LGs: MB_02 (2 QTL regions), 04 and 07. Of the QTL detected in the male map (Table 4 and Fig 2), two were expressed every year (2012–2016) and mapped to MB_02, in two different regions, and MB_07, explaining from 3.6 to 7.7% of the phenotypic variance (PV) for time of budburst. One QTL was detected only in 2016 on MB_04, explaining 4.9% of PV.

### Gene identification for time of leaf budburst

The genomic structure of the ‘major’ QTL detected on TGdL_02 in all five years included the SSR locus AJ417975b [29] and was investigated in detail. This QTL region contains 25 genes located in 5 scaffolds (Table 5). One of them (scaffold 1048, 19.8 cM) hosts the gene g8523.t1, which is the same orthologous gene that contains the mapped AJ417975b locus, which was placed at 19.2 cM in the female map and codes for a lipoxygenase 1(LOX1). By analyzing gene ontology terms of the 25 genes, some GO terms were noted to be enriched: GO:0044446 (intracellular organelle part) and GO:0005515 (protein binding) as discussed below.

**Table 5:** List of the 25 hazelnut genes, and relative functions, identified in the major QTL region for time of leaf budburst.

Hazelnut scaffold	cM	Hazelnut gene	Name	UNIPROT code	AGI code	e-value	Identity (%)	Similarity (%)
00690	17.2	Corav_g6384.t1	Gag-pol polymerase	B2BXI1	-	9.8e-18	27.2	55.0
00690	17.2	Corav_g6385.t1	Gag-pol3	B2BXM3	-	6.5e-35	37.7	65.4
00690	17.2	Corav_g6388.t1	Polynucleotidyl transferase, ribonuclease H-like superfamily protein	F4I526	AT1G56310	3.5e-231	61.6	80.3
00690	17.2	Corav_g6390.t1	Unknown protein, (DUF3511)	O22262	AT2G47480	1.46e-16	45.4	67.0
00690	17.2	Corav_g6391.t1	RECQ helicase l1 (RECQI1)	Q9FT74	AT3G05740	3.2e-232	63.8	79.0
00690	17.2	Corav_g6392.t1	GPI-anchored adhesin-like protein (TRM8)	Q6NQ74	AT5G26910	4.9e-101	39.2	62.6
00690	17.2	Corav_g6393.t1	Calmodulin-binding protein (DUF1645)	AR781	AT2G15760	9.8e-14	37.9	53.8
09783	17.3	Corav_g27951.t1	Inorganic H pyrophosphatase family protein (AVP1)	P31414	AT1G15690	0.00094	45.5	71.2
09783	17.3	Corav_g27952.t1	Inorganic H pyrophosphatase family protein (AVP1)	P31414	AT1G15690	0	86.3	94.6
04269	17.6	Corav_g19793.t1	Calmodulin-binding protein	Q0WVV6	AT4G25800	3.6e-69	39.9	63.6
00998	18.6	Corav_g8254.t1	C2H2 and C2HC zinc fingers superfamily protein (MGP)	Q9ZWA6	AT1G03840	1.2e-102	83.9	89.7
00998	18.6	Corav_g8255.t1	Photosystem II reaction center psbp family protein	F4J7A7	AT3G05410	9.0e-114	70.2	87.8
00998	18.6	Corav_g8256.t1	Jmjc domain protein (JMJ24)	F4HZD1	AT1G09060	0.0e-00	59.9	77.7
00998	18.6	Corav_g8257.t1	Acyl-coa binding protein 4(ACBP4)	Q9MA55	AT3G05420	1.4e-281	73.5	87.3
00998	18.6	Corav_g8258.t1	Tudor/PWWP/MBT superfamily protein	F4K4D6	AT5G27650	1.1e-226	53.7	70.2
01048	19.8	Corav_g8523.t1	Lipoxygenase 1(LOX1)	Q06327	AT1G55020	3.9e-153	73.3	89.4
01048	19.8	Corav_g8524.t1	Plant neutral invertase family protein (A/N-inva)	Q9FXA8	AT1G56560	3.7e-310	78.2	89.3
01048	19.8	Corav_g8525.t1	Pentatricopeptide repeat-containing protein	D7KB47	-	4.8e-238	65.7	82.2
01048	19.8	Corav_g8526.t1	Tetratricopeptide repeat (TPR)-like superfamily protein (OTP82)	Q9LN01	AT1G08070	7.0e-193	40.7	68.3
01048	19.8	Corav_g8527.t1	Photosystem II reaction center psbp family protein (PPD1)	O23403	AT4G15510	1.9e-99	77.0	89.2
01048	19.8	Corav_g8528.t1	Clathrin adaptor complexes medium subunit family protein (ZIP4)	F4I562	AT1G56590	6.2e-225	79.5	93.5
01048	19.8	Corav_g8529.t1	Chloroplast RNA binding protein (CRB)	Q9SA52	AT1G09340	3.7e-200	89.3	96.6
01048	19.8	Corav_g8530.t1	Galactinol synthase 2 (gols2)	Q9FXB2	AT1G56600	6.4e-118	76.5	88.7
01048	19.8	Corav_g8531.t1	Galactinol synthase 2 (gols2)	Q9FXB2	AT1G56600	4.3e-134	78.9	91.3
01048	19.8	Corav_g8532.t1	Invertase/pectin methylesterase inhibitor family protein	D7M341	-	7.2e-15	32.1	55.7

The QTL region (lb_TGdL_02) here showed is comprised between 17 cM and 20 cM in the TGdL map.

## Discussion

### Map construction

GBS has proven to be a rapid and efficient method to analyze whole genomes, enabling discovery of thousands of SNP. The restriction enzyme *Ape*KI, a five-base cutter, was used here for the construction of reduced representation libraries, to obtain high genome coverage. When *Ape*KI was used to reduce genome complexity in maize, the sequence tags obtained covered 2.3% of the genome [11]. In our case when we mapped cleaned reads against the Jefferson hazelnut genome (http://www.cavellanagenomeportal.com), we covered approximately 0.6% of the genome sequence. In this study, a total of 9,999 SNPs were discovered. This number is comparable to that found by Bushakra et al. (2015) [48] in black raspberry (7,911 SNPs) and by Bielenberg et al. (2015) [14], who identified 9,998 SNPs distributed across all major scaffolds of the peach genome. The frequency of SNPs was one in every 206 nucleotides, which is comparable to that found in rubber tree (1/308 nt) [9]. Out of the 9,999 SNP loci discovered, 7,343 (73%) segregated in the progeny and were evaluated for mapping. The double pseudo-testcross mapping strategy is considered the most suitable technique to construct genetic maps in outcrossing species; the efficiency of this strategy depends both on the level of heterozygosity of the species, and on the level of detectable polymorphism between the parents [49,50]. Hazelnut is well-suited because it is a highly heterozygous species, attributable in part to the sporophytic self-incompatibility that enforces cross-pollination [23]; moreover the level of polymorphism between our parents is very high, since TGdL and MB show several phenotypic differences. TGdL has an earlier phenology than MB (time of budburst, male and female flowering, and nut maturity); moreover the TGdL tree is less vigorous and more susceptible to big bud mite (*Phytoptus avellanae*) than MB, and finally the two cultivars differ for nut traits (size and shape, percent kernel).

Of the total 7,343 segregating SNP loci, only those that segregated within one of the parents were chosen for mapping. Intercross markers which segregated with a 1:2:1 ratio were excluded, since the inclusion of markers segregating within both parents produce an estimate of recombination frequency which is the average of both male and female meioses, and may differ from testcross frequencies. Therefore conflicts can arise between marker orders [51].

Since the use of falsely discovered SNPs for the construction of linkage maps could result in low-quality genetic maps, we applied further stringent criteria to filter SNPs suitable for mapping. We excluded SNPs with highly significant distortion or identical segregation patterns. In addition, we considered only markers grouped at a LOD score of 12.0 and, to avoid redundancy, one marker per scaffold. Of the 7,343 total segregating SNPs, 1,216 (16.5%) and 1,192 (16.2%) were used for the construction of the parental maps. Such numbers are comparable and even higher than the ones reported in previous studies in other species. In sweet cherry, of 8,476 segregating selected SNPs, 443 (5.0%) and 474 SNPs (5.6%) were used for mapping the two parents [52]. In blueberry, 17,846 SNPs were identified but only 785 (4.4%) and 450 (2.5%) were used for mapping the parents [53].

On the female map, 111 of the SNP and SSR (9.0%) markers showed marginal segregation distortion, while on the male map this value was 70 (5.8%). Since segregation distortion frequently leads to a significant under- or overestimation of recombination fraction [54], to reduce the probability of false linkage, only markers with marginal segregation distortion, deviating up to 1% [49,55] were retained, while markers with significant segregation distortion were excluded for mapping. In this work, SSR loci were chosen in order to facilitate the identification and to assign the progressive number to each linkage groups, accordingly to Beltramo et al. (2016) [29] map order.

The construction of the female and male parental maps using the selected markers yielded two maps of comparable length and average density: 900.4 cM and 0.8 cM for TGdL and 899.1 cM and 0.8 cM for MB (Table 2). Except for a few distal regions on some linkage groups, markers were distributed uniformly on both the TGdL and MB maps. Only eight gaps in the TGdL map and five gaps in the MB map were larger than 5 cM.

We implemented the previous framework map [29], by dramatically increasing maps length and density (about 900 cM and 0.8 cM, against 663.1 cM and 4.45 cM, respectively) thanks to the use of high-throughput next-generation sequencing technologies. This was due to the use of bi-allelic markers (SNP), which even if are less polymorphic than SSRs, they are more abundant, genome-wide distributed and mostly derived from genes [56]; moreover they can be easily converted into a single-locus marker method of detection in a fast reliable and low cost way; as example, a simple and economical genotyping method is Tetra-Primer ARMS-PCR [57], which involves a single PCR followed by gel electrophoresis.

### Bioinformatic approach to disentangle ‘pseudo-linkage’ in reciprocal translocation event

When the TGdL map was developed, linkage was found of markers belonging to TGdL_09 and TGdL_10. This was indicative of a reciprocal translocation event. Translocations are well-documented in various crops including *Prunus* [58], soybean [59], rye [60], and barley [61]. In hazelnut, cytogenetic analysis showed that the decreased pollen fertility (40–70%) observed in some cultivars, including Barcelona (syn. Fertile de Coutard), Negret, Tonda di Giffoni, Segorbe and TGdL, is related to the presence of reciprocal translocations [18,19]. Although only half of the pollen is expected to be viable due to the unbalanced gametes that result from meiosis, Salesses and Bonnet (1988) [19] suggest that the productivity of these cultivars is not appreciably reduced. However, abnormalities like this are significant in genetic studies, especially in a breeding program, and reciprocal translocations complicate the construction of genetic maps [62].

Applying the technique of Farré et al. (2011) [21], we were able to dissect ‘pseudo-linkage’ between markers, discarding those very close to the translocation breakpoints and which exhibited severe segregation distortion. We were thus able to generate a linear and unambiguous sequence of markers for each of the two chromosome arms and for each translocated LG. In order accurately to utilize this technique we depended absolutely on the availability of a detailed and high resolution map of MB, a cultivar which does not contain the translocation. It was only because of this map that we were able to identify the translocation points and denote markers on the chromosome arms involved. On the basis of markers on common scaffolds in TGdL and MB, the position of translocation breakpoints was determined to be 47 cM on LG_09 and 23 cM on LG_10, corresponding to the positions of scaffolds Sc.AJ_01333 and Sc.AJ_04559 on the MB map. In order to characterize the translocation breakpoint, we identified 101 plants in the F_1_ population that carry the translocation (TGdL type) and 92 with the pre-translocation chromosome architecture (MB type). Twenty individuals were discarded because they could not be classified in either of the two types due to deviating chromosomal combination. Some of these deviations concerned so-called ‘singletons’; possibly arising from observation errors or due to gene conversions, as reported by Farré et al. (2011) [21]. The arbitrary chromosome arms obtained were designated TGdL_09a, 09b, 10a and 10b (Fig 2). A genetic consequence of reciprocal translocation is that linkage relationships in a translocation heterozygote are altered as recombination between loci may be significantly reduced, particularly within the interstitial segment and between genes close to the translocation breakpoints [21]. For future breeding and to fully understand the relevance of this genetic modification, it is worthwhile to follow individuals carrying interchanged chromosomes and to expand the study on the occurrence of reciprocal translocation to a wider range of cultivars, examining the effect on pollen and embryo sac fertility. Salesses (1973) [18], and Salesses and Bonnet (1988) [19] observed reduced pollen fertility but considered yield to be unaffected by the meiotic abnormality. This may be due to the presence of several embryo sacs within the hazelnut ovule [63] or to other reasons, such as gene duplications in the genome, but these authors also observed translocations between different non-homologous chromosomes in different cultivars. While Barcelona and Tonda di Giffoni are recognized as high-yielding cultivars, TGdL is known to have lower yield with year-to-year variations. Further investigations could clarify whether this is just due to environmental factors or has a genetic basis related to the reciprocal translocation.

### QTL mapping for time of leaf budburst

The presented high-density maps dramatically enhance the possibility for marker-assisted selection (MAS) in hazelnut. Hazelnut breeding is difficult and time consuming due to its self-incompatibility, long juvenile period and high heterozygosity. MAS could identify young seedlings with favorable alleles or desirable traits [64,65]. As a demonstration, we provide an in-depth QTL analysis for the time of leaf budburst in the same progeny, previously analyzed by Beltramo et al. (2016) [29], but increased in the number of individuals. Time of leaf budburst is a very important trait that can limit the expansion of the cultivation to new growing regions with climate conditions that differ from those of the traditional areas. Bud phenology is under strong genetic control [66,67], however this trait is also environmentally driven by photoperiod and temperature [68,69,70]. Differences in chilling and heat requirements, necessary to overcome dormancy in temperate zones, are genetic adaptations to environmental conditions [70]. The requirement for chilling is an adaptive device for preventing the beginning of leafing in the winter (e.g. during transient warm events), when leaves are likely to be damaged by frost [71,72]. Chilling and heat requirements differ from cultivar to cultivar and different parts of the hazelnut plant including catkins, female flowers and leaf buds, have different requirements. Cultivars of Turkey and southern Italy have lower chilling requirements than those that originated in central Europe. Our female parent, TGdL, originated in Italy and needs approximately 760–860 CH (Chilling Hours, calculated by counting the number of hours between 0–7°C) to leaf out, while the male parent MB, native to central Europe, needs 990–1040 CH [73]. In our progeny, the time of leaf budburst showed, across the five years of observations, a broadly normal (or Gaussian) distribution. Transgressive segregation was observed, with a few individuals showing phenotypes more extreme than the parents (Fig 3). The possibility to have individuals with very early or very late time of leaf budburst, improves the opportunities of commercially growing hazelnut in areas with climatic conditions different from traditional ones. In fact, the chilling requirement of vegetative buds is a major consideration in determining the area of cultivar adaptation [73]. Plants with late time of leaf budburst are likely less susceptible to the spring frost and could be more suitable for cold environmental conditions. On the other hand, genotypes with early leaf budburst could be more suitable to warm environmental conditions, where they can have early nut maturity, a very desirable trait for producers and industrial users. QTL mapping is a powerful method to identify genomic regions controlling this trait. The first report of QTL influencing leaf budburst was in poplar in 1995 [67], followed by Douglas fir [74], chestnut [75], oak [76,77] and, more recently, willow [78]. Using the highly saturated linkage maps, we identified QTL for budburst in each year of observation. In particular, we identified 11 QTL on the male and 18 QTL on the female maps (Table 4). Collard et al. (2005) [79] suggested that a QTL can be classified as ‘major’ if it can account for > 10% of the PV. A more enhanced definition of ‘major’ requires that the QTL be stable across multiple seasons or locations [80,81]. Based on these criteria, the most interesting QTL region we found is on TGdL_02, as it was detected in all five years and explained from 31.4% (year 2012) to 54.6% (year 2016) of the PV. This result is not surprising, since it was already described by Beltramo et al. (2016) [29], using a subset of the same hazelnut progeny in three years of observation. Finally, we found other ‘minor’ QTL scattered in different LGs, not surprising since the regulation of phenology is quite complex and involves different pathways [82].

With the increase in the number of years of observation and thanks to the GBS approach we were able to better define the genomic region underlying the time of budburst, identifying new SNP markers and genomic regions not previously identified, strictly associated to QTL for time of leaf budburst; in fact in addition to the major QTL on TGdL_02 already described [29], we have identified several new QTL. This result may help to identify genes controlling seasonal budburst in hazelnut, at least in the progeny used for mapping, as described in the next paragraph.

### Investigation of candidate genes involved in regulation of time of budburst

On the female map (TGdL), the region on LG_02 between 17 cM and 20 cM (Fig 2) is particularly relevant for determining time of leaf budburst. This QTL is associated with SNP markers (Sc.AJ_00690_17369, Sc.AJ_09783_7017, Sc.AJ_04269_19964, Sc.AJ_13518_3809, Sc.AJ_00998_24907, Sc.AJ_01048_1235) lying in 6 scaffolds of the Jefferson genome. These scaffolds were studied to detect gene functions of interest likely related to budburst and, overall, 25 genes were spotted. The QTL is associated with the SSR locus AJ417975b, an EST-SSR derived from a *Betula* sequence of the Lox gene [29]. In the scaffold 01048, placed at 19.8 cM in the female map, one of the genes (Corav_g8523.t1) is the same Lox gene associated with the SSR locus AJ417975b, but placed at 19.2 cM in the female map. This map distance discrepancy is not surprising since the SSR locus was placed in the female map using an intra-gene polymorphism segregating in the progeny, whilst the Corav_g8523.t1 map position corresponds to the position in the map of scaffold 01048, identified by GBS data using markers not positioned within the target gene. The gene Corav_g8523.t1 (i.e.: AJ417975b) codes for a lipoxygenase (LOXs). LOXs are a class of dioxygenase that catalyze the oxygenation of polyunsaturated fatty acids to form many biologically active compounds, involved in several physiological processes, with diverse functions. These functions include the regulation of plant growth and development, such as potato tuber growth [83], soybean leaf development [84], corm development [85], almond seed development [86] and response to biotic and abiotic stresses [87,88]. Given the presence of multiple isozymes of lipoxygenase plants, it is possible that individual lypoxigenase isozyme within a plant, may have distinct physiological roles [84], including role likely related to the budburst phenotype.

In the same scaffold (01048), two interesting genes were present (Table 5). The first gene (A/N-InvA, Corav_g8524.t1) encodes for a mitochondrial neutral/alkaline invertase that cleaves sucrose into glucose and fructose. In general, high glucose levels favor cell division and expansion, suggesting that these proteins are involved in overall plant development. Moreover, generated glucose can be used as a substrate for mitochondria-associated hexokinase (mtHXK), contributing to mitochondrial reactive oxygen species (ROS) homeostasis, suggesting a role of these proteins in the oxidative stress defense [89]. The second gene (CRB, Corav_g8529.t1) encodes for a chloroplast RNA-binding protein (CRB) that in *Arabidopsis* is supposed to have a role in transcript regulation in the chloroplast. This gene is associated with pre-ribosomal particles in chloroplasts, and participates in chloroplast ribosomal RNA metabolism, probably during the final steps of 23S rRNA maturation. It binds and cleaves RNA, particularly in stem-loops and may enhance transcription by the plastid-encoded polymerase and translation in plastid via the stabilization of ribosome assembly intermediates. It is likely involved in the regulation of the circadian system. Mutations in CRB have profound effects on the chloroplast morphology and photosynthetic performance, as well as on the functioning of the circadian system [90].

Besides the described genes, some enriched GO terms were highlighted: GO:0044446 (intracellular organelle part) and GO:0005515 (protein binding). The latter enriched term contained some regulatory proteins such as Corav_g8254.t1, Corav_g8256.t1 and Corav_g19793.t1. Corav_g8254.t1 (MAGPIE—MGP, scaffold 00998; 18.6 cM), which codes for a zinc finger-like protein that in *Arabidopsis* controls SHORT-ROOT (SHR) and SCARECROW (SCR) transcription factors activity in a transcriptional/protein interaction network [91] and regulates tissue boundaries and asymmetric cell division. A scarecrow-like transcription factor 6 (SCL6), a member of the GRAS gene family and controlling a wide range of developmental processes including hormone signaling and bud formation, was found and described in grapevine [92]. Corav_g8256.t1 (scaffold 00998), is a jumonji domain-containing protein involved in chromatin DNA binding, having a positive regulation of gene expression. Corav_g19793.t1 is a calmodulin-binding protein, a transcription activator that binds DNA in a sequence-specific manner, to promote the expression of target genes.

## Conclusions

This paper is the first report concerning the construction of well-saturated hazelnut genetic maps using SNP markers and its application for phenological related traits QTL analysis. The high-density maps described here establish a backbone that will facilitate future gene localization for hazelnut. This work confirms, based on linkage of markers, that a reciprocal translocation is present in hazelnut TGdL as the result of an interchange of chromosome arms between LG_9 and LG_10. A ‘major’ QTL on LG_02 for time of leaf budburst explaining 30–50% of the PV was described and represents a promising tool for hazelnut breeding. Future work will focus on functional studies of candidate genes, and on the search of allelic differences within this QTL region that are related to different times of budburst in order to develop markers for MAS.

## Supporting information

S1 File Complete list of all SNPs identified in this study. SNPs are categorized in five types: I) both parents homozygous, monomorphic; II) both parents homozygous, polymorphic; III) both parents heterozygous, monomorphic, IV) TGdL heterozygous, MB homozygous; V) TGdL homozygous, MB heterozygous.
(XLSX)

S2 File Accessory SNP markers. A full list of additional accessory SNP markers for TGdL and MB, excluding from map construction because they were in excess since only one marker per scaffold was chosen.
(XLSX)

S3 File Detailed genetic maps of the *Corylus avellana* cultivars Tonda Gentile delle Langhe (TGdL) and Merveille de Bollwiller (MB). TGdL (female parent, blue LGs on the left) and MB (male parent, yellow LGs on the right), and location of QTL responsible for time of leaf budburst. Homologues LGs are presented side-by-side and aligned on the base of markers developed on common scaffolds, here connected with a line. For the female map, marker names are shown on the left of each linkage group, for the male map the mirror arrangement applies. Markers showing significant levels of segregation distortion are indicated by *asterisks* (*: 0.1 >*P ≥* 0.05, **: 0.05 >*P ≥* 0.01). Loci mapped in repulsion phase are flanked by an “-r”. The QTL bar represents the region above the genome-wide LOD threshold. The left rulers expressed the length of the LGs and the QTL position in cM.
(PDF)

S4 File LOD curves for the major QTL responsible for time of leaf budburst detected on TGdL_02 in five years of observations (2012–2016). The estimated LODs at the QTL peak is reported and the dashed line indicates the genome-wide LOD Thresholds (GW) as determined by a permutation test at p *≤* 0.05.
(TIF)
