# Supplementary figures and images for: High density SNP mapping and QTL analysis for time of leaf budburst in Corylus avellana L
Source: PLoS One. 2018 Apr 2;13(4):e0195408. doi: 10.1371/journal.pone.0195408 (PMC5880404; doi:10.1371/journal.pone.0195408)

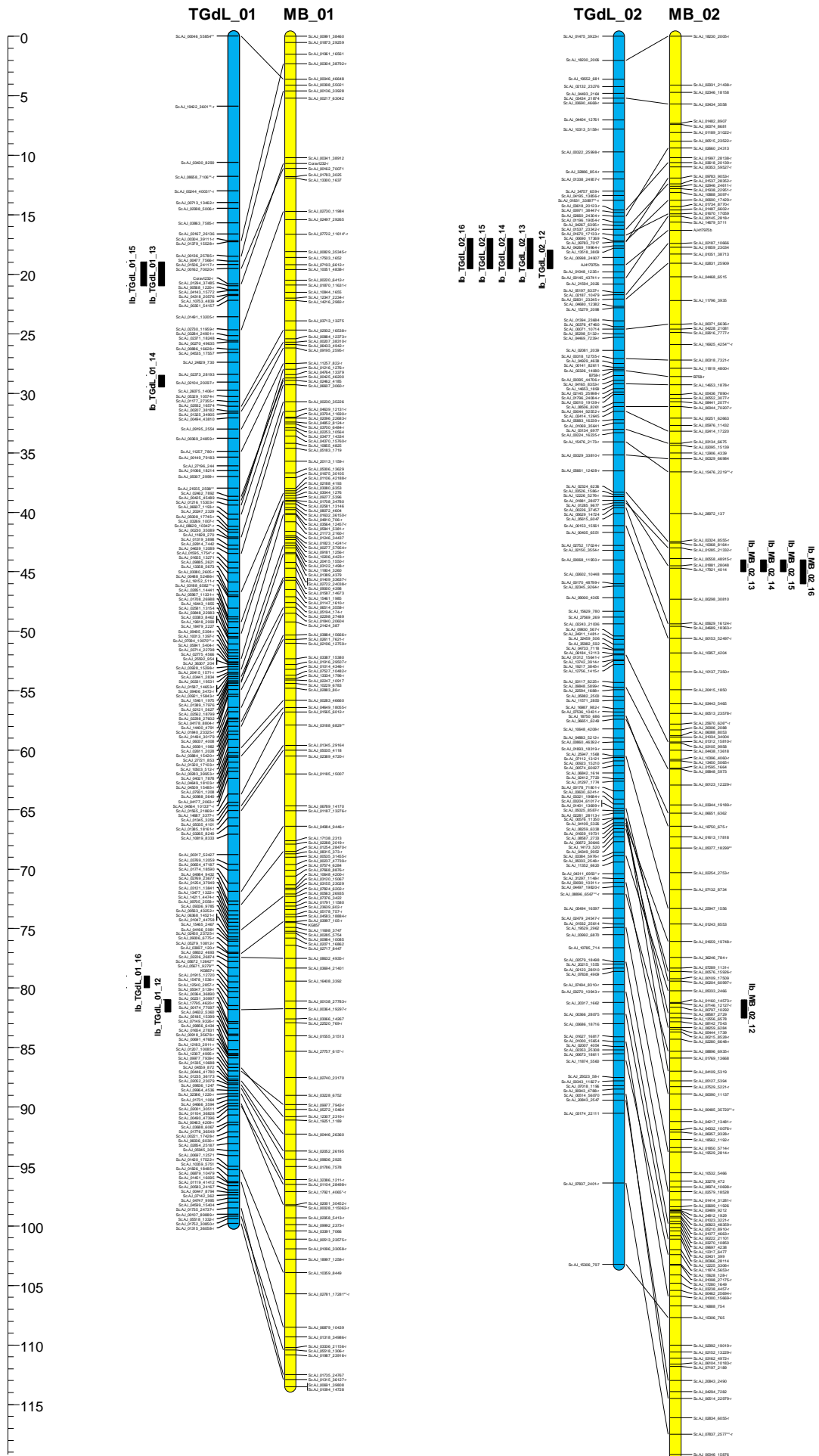

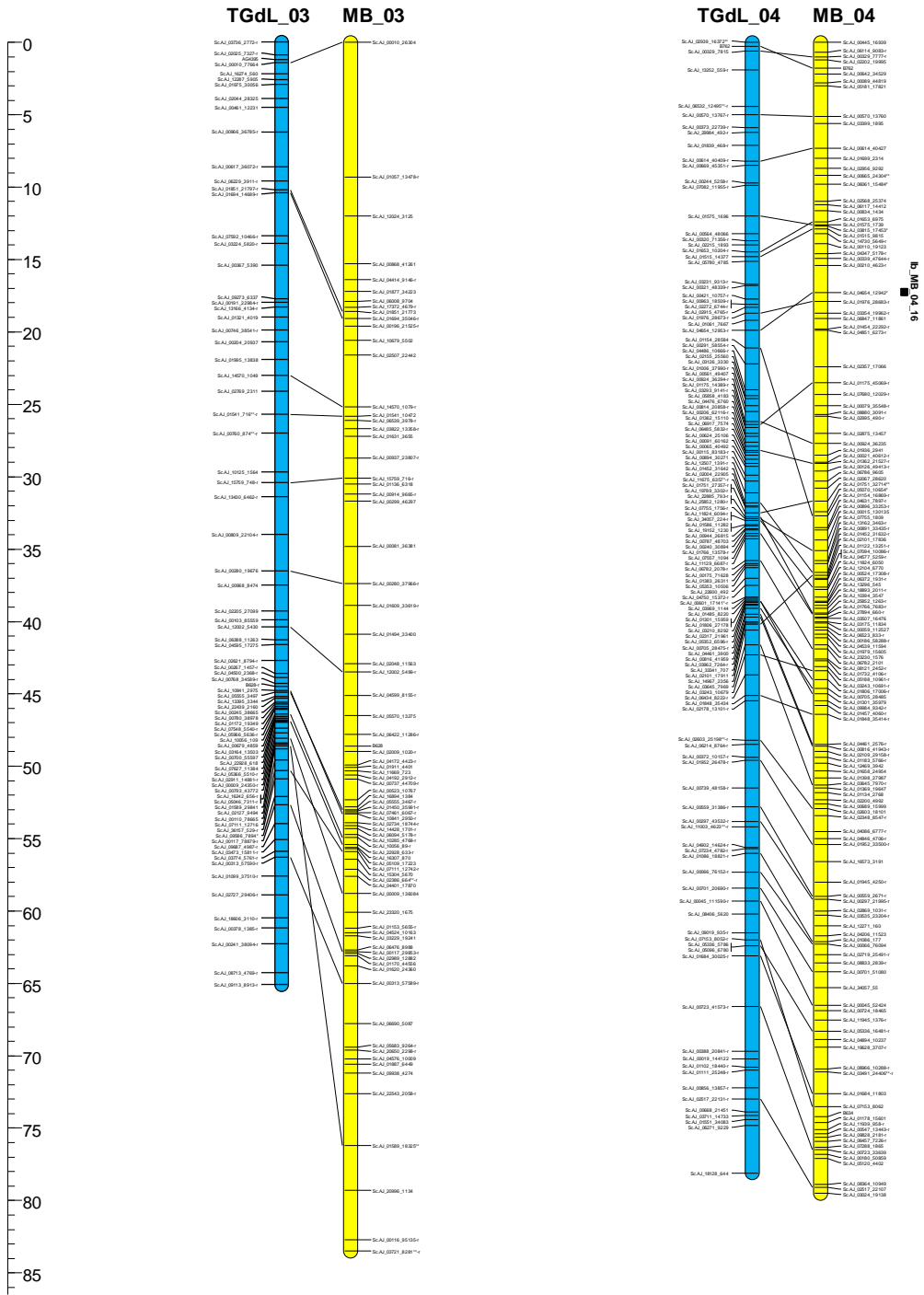

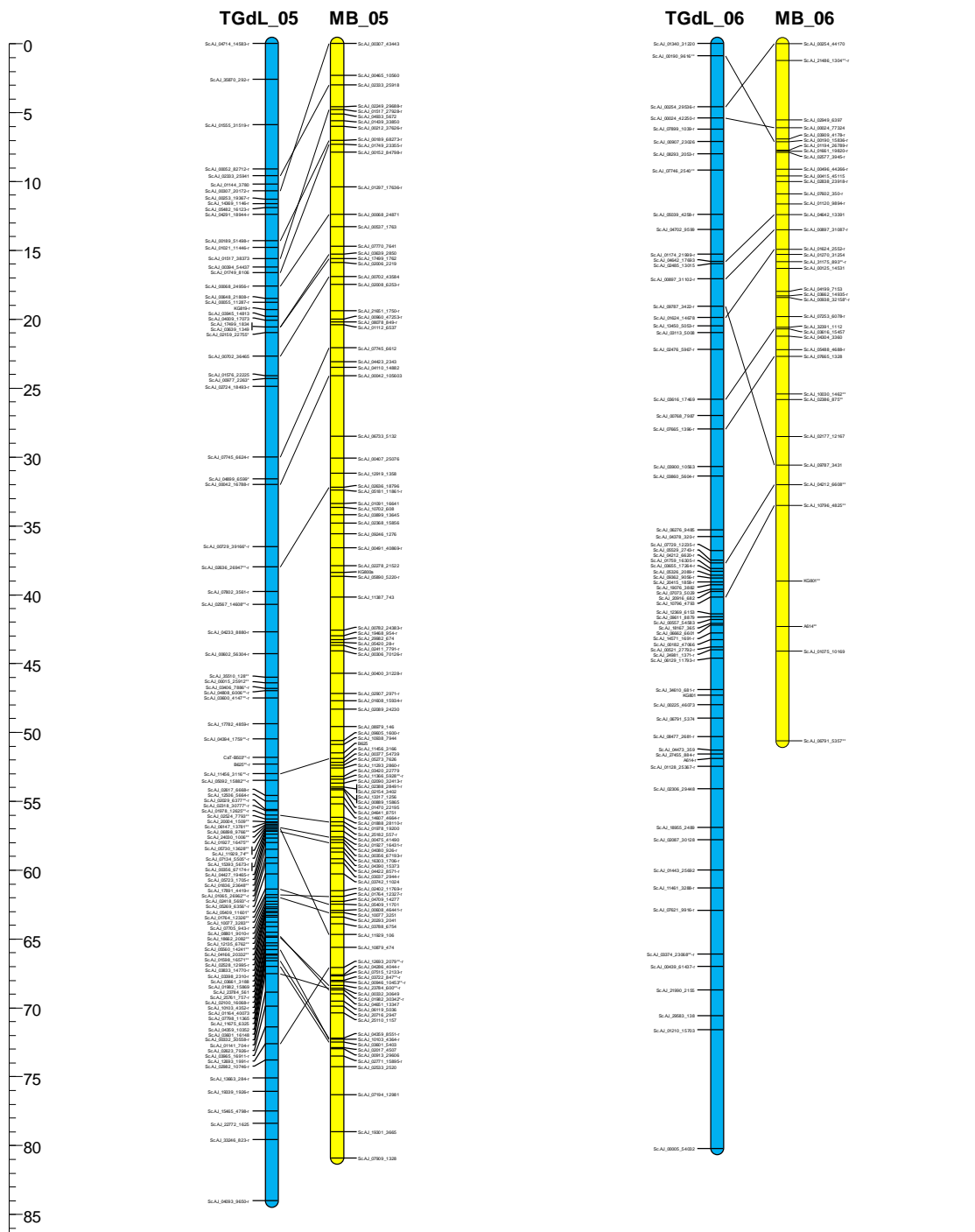

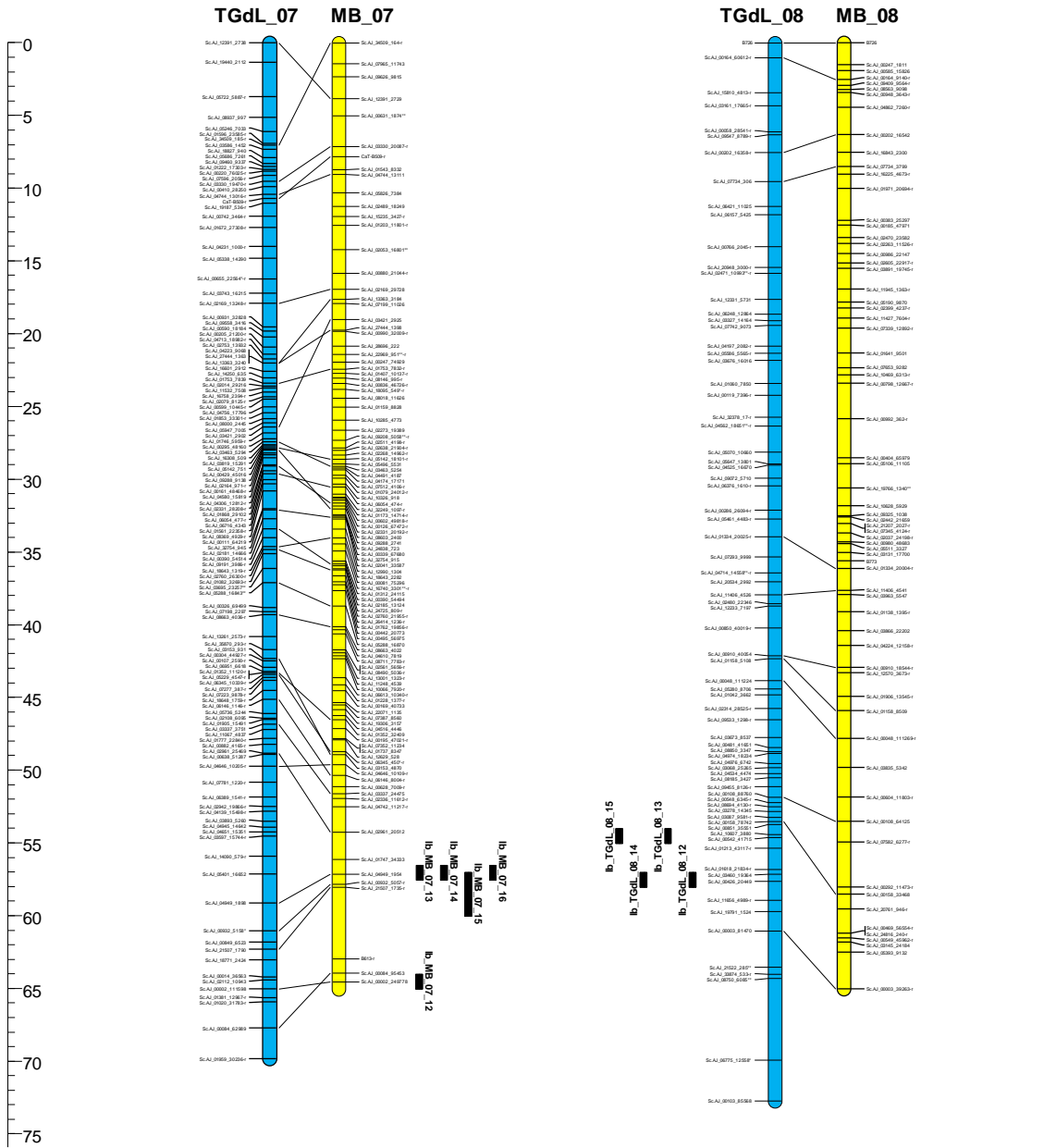

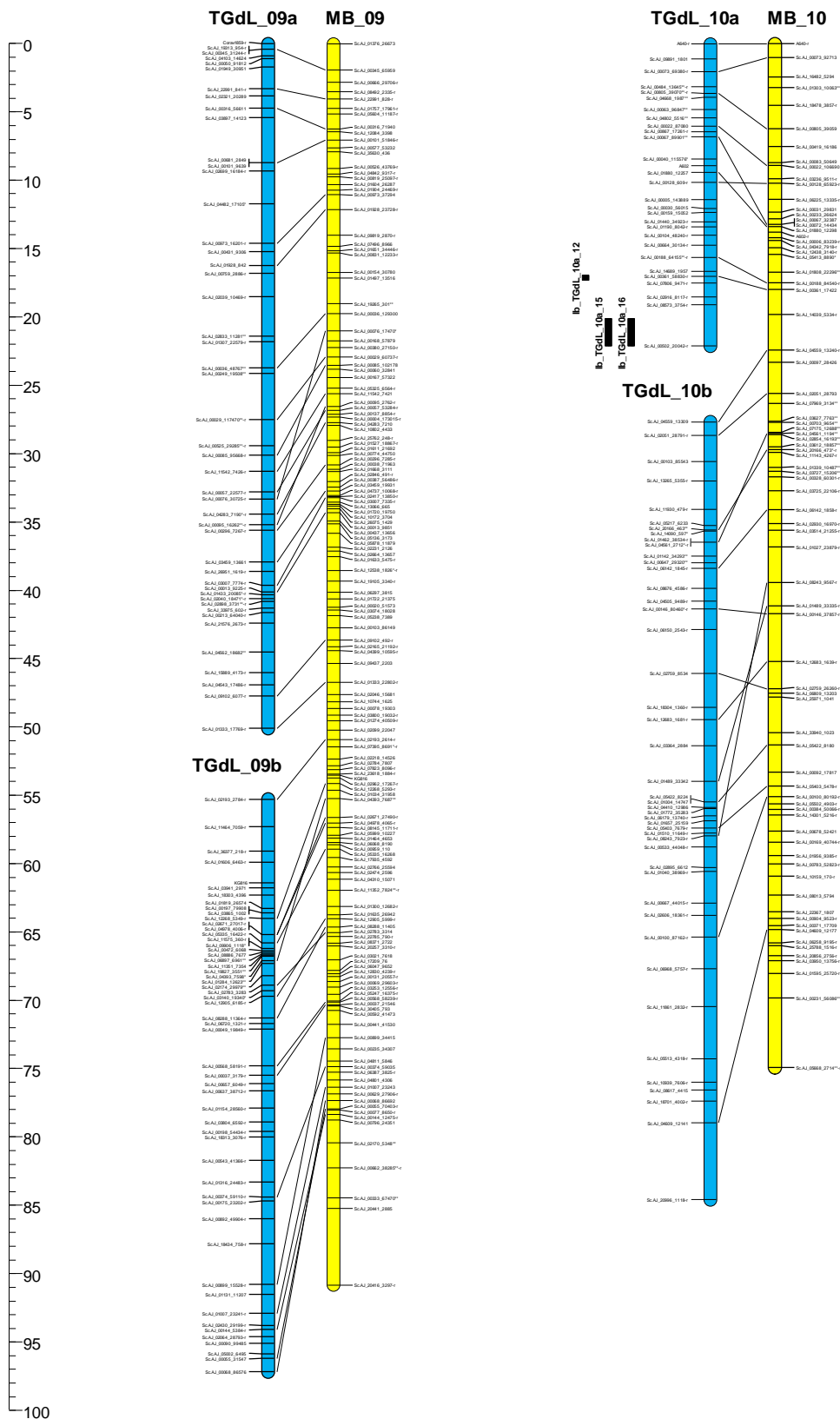

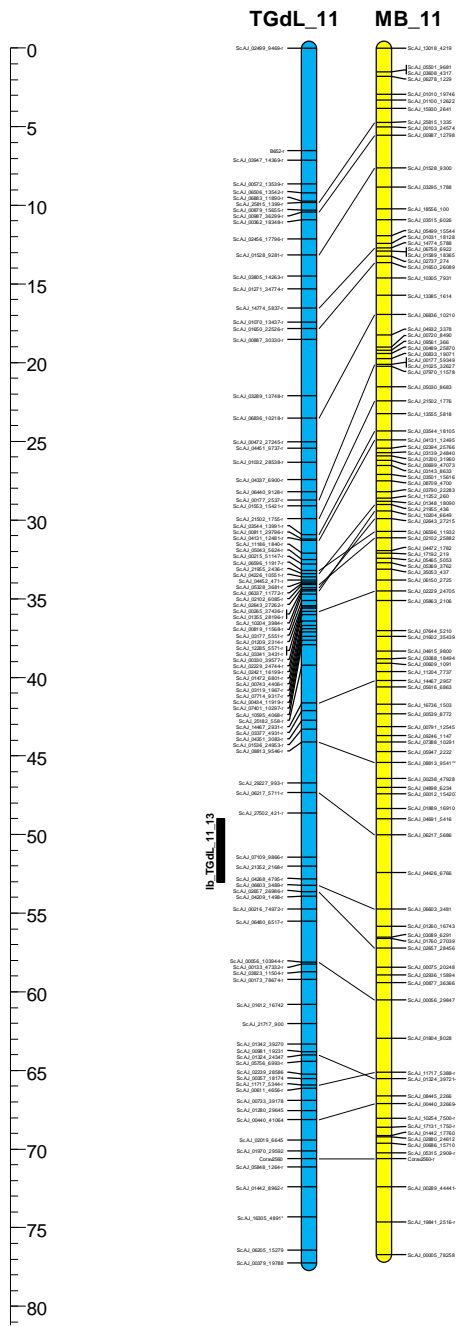

Supplement: S3 File — TGdL (female parent, blue LGs on the left) and MB (male parent, yellow LGs on the right), and location of QTL responsible for time of leaf budburst. Homologues LGs are presented side-by-side and aligned on the base of markers developed on common scaffolds, here connected with a line. For the female map, marker names are shown on the left of each linkage group, for the male map the mirror arrangement applies. Markers showing significant levels of segregation distortion are indicated by asterisks (*: 0.1 >P ≥ 0.05, **: 0.05 >P ≥ 0.01). Loci mapped in repulsion phase are flanked by an “-r”. The QTL bar represents the region above the genome-wide LOD threshold. The left rulers expressed the length of the LGs and the QTL position in cM. (PDF) [file pone.0195408.s003.pdf]

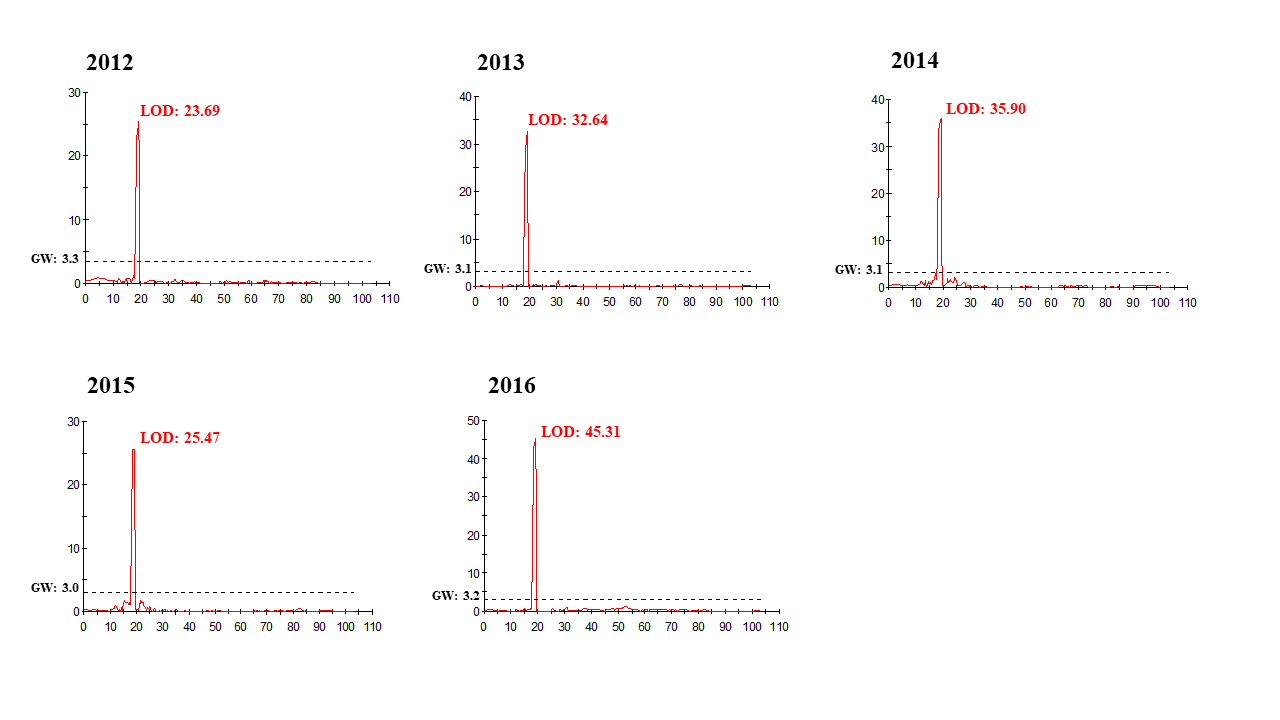

Supplement: S4 File — The estimated LODs at the QTL peak is reported and the dashed line indicates the genome-wide LOD Thresholds (GW) as determined by a permutation test at p ≤ 0.05. (TIF) [file pone.0195408.s004.tif]
